# Clinical Reasoning as the Guiding Framework in Medical Biochemical Test Reporting

**DOI:** 10.3390/diagnostics16152475

**Published:** 2026-08-05

**Authors:** Torleif Trydal, Geir Erland Tjønnfjord

**Affiliations:** 1 Department of Clinical Research, Sørlandet Hospital Trust, 4604 Kristiansand, Norway; 2 Department of Haematology, Oslo University Hospital, 0424 Oslo, Norway; gtjonnfj@ous-hf.no; 3 Institute of Clinical Medicine, University of Oslo, 0318 Oslo, Norway

**Keywords:** clinical chemistry, classification, reference values, bias, harmonization, ferritin, postmenopause

## Abstract

Clinical biochemical reports should reflect the clinical perspective, be well organized and biologically oriented, and present accurate reference values based on clinically relevant distributions. To illustrate possible quality issues, we selected Clinical Cases from *The New England Journal of Medicine* as it publishes comprehensive reports to teach internationally relevant high-level workups. We evaluated their organization with a system organ grouping and the consistency of reference values between comparable cases. We found that the ordering of tests varied across issues, and associated interpretative tests were not organized in a scholarly manner. In several cases, the reference values for hemoglobin did not align with WHO recommendations, the upper reference value for glycohemoglobin did not correspond to established guidelines, and the upper reference value for creatinine did not reflect internationally standardized methods. The ferritin reference values in young women were not adjusted for the postmenopausal age group. When searching for incident blood loss using a common reference in young women and those of postmenopausal age in a large primary health care cohort, the frequency of flagged low ferritin results was so low that the ratio of flagged results to the incidence rate of colorectal cancer was only 1.1 (0.95 confidence interval 1.0 to 1.3). In men, low ferritin flagging was far more prevalent, resulting in a ratio of 11.7 (0.95 confidence interval 11.2 to 12.1). Thus, men had an 11-fold higher theoretical relative sensitivity when detecting occult iron loss compared with women. System organ assessment is a basic clinical competence, and system organ classification for clinical biochemical tests could be used to organize clinical laboratory reports. The reference values provided should be biologically based, harmonized, and readily recognizable by the clinician to accurately guide decisions, in contrast to local procedural determinations. Examination of local laboratory data may reveal biased laboratory support.

## 1. Introduction

Laboratory support and clinical laboratory reports are cornerstones of clinical workups. Clinical laboratory reports are expected to reflect a scholarly organized approach that complements clinical workups. Furthermore, the reference values are the anchor for interpreting a person’s results and are supposed to reflect the unbiased biological distribution of a presumed healthy population. These reference values are effectively fixed calibration points from which clinical decisions are made. Therefore, clinicians need standardized and harmonized analyses with precise reference values that are common, recognizable, and independent of various laboratories or practices. The essence is that clinicians weigh normal and abnormal test results relative to the reference value in different clinical scenarios. However, establishing accurate and robust reference values from truly healthy individuals with clinically meaningful stratification is not only challenging but may be impossible given the magnitude of variables that must be assessed, such as sex, age, pregnancy, environmental factors, genotypes, phenotypes, and ethnicity. Therefore, the basis for reference values should be published and readily available to users.

Clinical symptoms and signs often reveal disturbed biochemical homeostasis. The results from biochemical tests can be regarded as confined glimpses into composite and dynamic biology. The tests show fragments of interdependent biochemical functions and provide insights into normal or pathological biology. The term “biomarker” is increasingly used in the published literature. In total, 32% of articles in PubMed containing the term “creatinine/blood” also contained the term “biomarker” during 2021 and 2022 [National Center for Biotechnology Information, U.S. National Library of Medicine; Support 19 September 2023], reinforcing the notion of test measures as indicators of disease. It could be argued that there are no markers as such in the body, only biochemical quantities (measurands) that support a multidimensional interpretation of normal and pathological biology. We will draw attention to a systematic clinical approach to assessing normal and pathological states, emphasizing an integrated biological understanding.

To illustrate our case, we selected some of the most used biochemical tests by evaluating Clinical Cases from *The New England Journal of Medicine* 2021–2022, since the journal regularly presents comprehensive laboratory tables and these cases teach internationally relevant high-level workups. Published literature and data from a primary health care database were utilized to pinpoint some potential unfortunate routines or consequences in general clinical practice. We propose a consistent routine for reporting clinical biochemical tests. Local laboratory data and the reference values used should be examined to reveal possible bias in clinical laboratory support [1,2]. We want to draw attention to the fact that common practices may unnecessarily put patients at adverse risk.

## 2. Organization of Clinical Biochemical Reports

The laboratory tables were not consistently organized. The ordering of tests varied, and tests that are interpretively associated were not grouped together, such as major tests in hematology, immunology, endocrinology, blood-gas regulation, renal function, and drug measurement (Table 1). The grouping varied from issue to issue.

## 3. System Organ Classification

**Figure 1:**
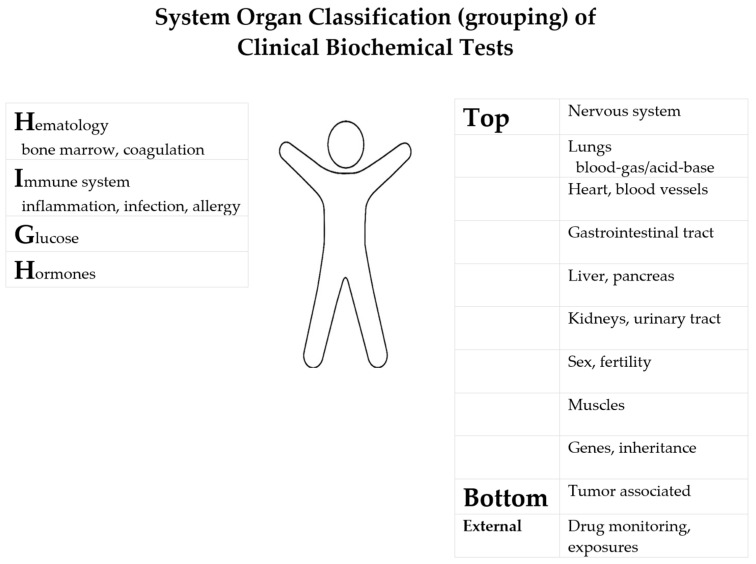
System organ classification (grouping) of clinical biochemical tests. A recommended starting point is the systems of hematology, immune system, glucose and hormones (mnemonic “HIGH”), followed by the organs “top to bottom”, similar to the system organ approach used in clinical work. Each test is listed once, in its preferred group. Because tests relate to various systems and organs, interpretation should follow a multidimensional or multiaxial elaboration involving all relevant systems and organs to construct a holistic picture. The tests could be divided into basic and extended categories. Specific reference to separate schemes may be beneficial (for example, microbiological, allergy, or intensive care unit schemes) using a computer-aided advanced user interface. Details are provided in Supplementary Sections S1–S4, also which includes selected recommended tests according to “The third WHO model list of essential in vitro diagnostics (EDL 3)”, *WHO Technical Report Series*, 2020;1031.

The organization of biochemical tests could be based on a system organ classification scheme, which focuses on enhanced biological understanding. Each test could be assigned to sections hematology, immune system, glucose, and hormones, and “organs top to bottom” completed by sections of genetic/inherited conditions, tumor-associated conditions, and external expositions including drug monitoring as appropriate (Figure 1, Supplementary Sections S1–S4).

A given test should be presented in only one section, thereby easing access to the result. A test should be associated with the group in which the analytical test has a major contribution to the biological status evaluation. The placement of a test should be clinically meaningful, not based on tradition or chemical characteristics. Most analyses, such as clinical observations, are not system-organ-specific, which requires multidimensional or multiaxial elaboration. The results from each item should therefore be interpreted in the context of available clinical and biochemical information to construct a holistic picture. The selection of tests in each practice is debatable and should evolve. However, the World Health Organization (WHO) recommends essential tests, which can be organized as basic and extended tests adapted to national policies [3] (Supplementary Sections S1–S4).

## 4. Consistency of Reference Values

Hemoglobin in whole blood, ferritin in serum, hemoglobin A1c in whole blood, creatinine in serum, and albumin in serum were selected to evaluate the consistency of reference values (commonly cited as “reference range” or “normal values”). The reference values given in the laboratory tables showed substantial variation among comparable cases and throughout a patient’s course (Table 2).

## 5. Non-Harmonized Reference Values and Potential Adverse Effects Caused by Bias

Universal standardization of hemoglobin measurement in whole blood is available, and WHO has published well-founded hemoglobin levels for the diagnosis of anemia in pregnant and non-pregnant women and men [4,5]. Inconsistencies blur universal interpretation. Unstandardized reference values impede possible individual adjustments attributable to age, different pregnancy stages, residential altitude above sea level, severity of anemia, some lifestyle habits, or possible hemoglobin genotypes [5,6,7].

**Figure 2:**
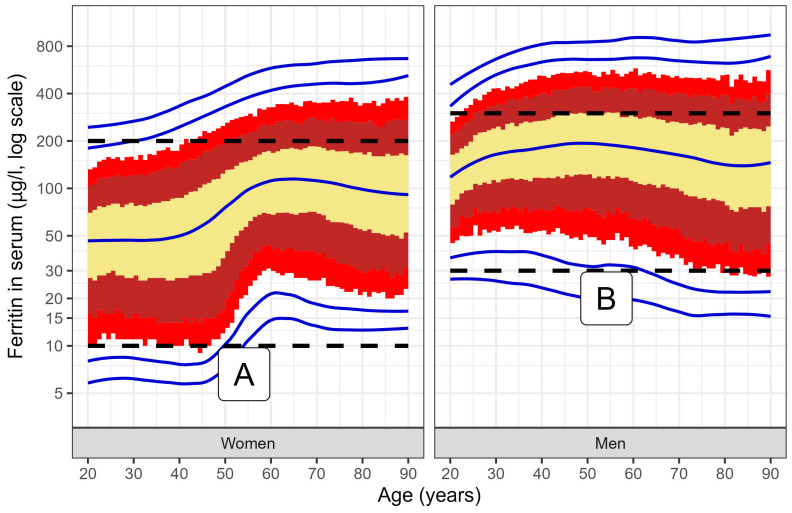
Ferritin results in serum from a primary health care cohort using the Roche ECLIA ferritin method (Roche Diagnostics, Basel, Switzerland). From top to bottom, the blue lines (smoothed) and the ribbon borders are the percentiles: 99 > 97.5 > 95 > 90 > 75 > 50 > 25 > 10 > 5 > 2.5 > 1. The dotted lines are the reference values: the lower reference values of 10 and 30 µg/l for women and men, respectively (as used by laboratories [2], and as cited in the handbook Massachusetts General Hospital Homepage, accessed 8 January 2021; the method used was Roche ECLIA, personal communication, 6 July 2022). Because the reference values were not adjusted in women for the postmenopausal change, the fraction of low ferritin values brought to attention by flagging was different between women and men after the age of 50–60 years (A and B in the figure). The clinical database was from the Department of Medical Biochemistry, Sørlandet Hospital Trust, Norway, selecting the first patient result per year during 2018–2021 (*n* = 302,929; 60% women); most participants were Caucasian. Statistical calculations and graphics were performed using R version 4.5.2 software with the data.table and ggplot2 packages, performing smoothing with non-linear loess regression (https://www.R-project.org) and RStudio 2025.09.2, Boston, MA, USA).

The lower reference values for ferritin in women did not mirror the normal biological increase in the ferritin concentration after menopause [2,8,9]. The omission of postmenopausal correction could lead to biased follow-up compared to men. In our own database comprising an unselected primary health care cohort aged 60–90 years, only 0.3% of women had results flagged for low ferritin values, in contrast to 3.8% of men, using lower reference limits of 10 µg/l common for young and older women and 30 µg/l in men (Figure 2. Roche ECLIA method; details in the figure legend).

When searching for incident blood loss in the postmenopausal age group in a Norwegian primary health care cohort, the ratio of flagged results compared to the incidence rate of colorectal cancer was only 1.1 (0.95 confidence interval 1.0 to 1.3; detailed results in Supplementary Material B). Low ferritin values in men were flagged far more frequently, resulting in a ratio to colorectal cancer incidence rate of 11.7 (0.95 confidence interval 11.2 to 12.1), indicating an 11-fold higher theoretical relative sensitivity compared with women for detecting bleeding cancer before possible iron-deficient anemia appears. Accounting for the biologically postmenopausal increase in ferritin level using lower reference values of 20 µg/l or 30 µg/l, respectively, 10.7 (0.95 confidence interval 10.2 to 11.1) and 26.2 (confidence interval 25.6 to 27.0) had flagged measurements relative to the colorectal incidence rate; thus, the ratio of men to women were 1.1 and 0.4, respectively. Changed biological distributions may be acknowledged when clinically relevant, as in this case, which objectively increases the sensitivity of detecting occult bleeding disorders. Diagnostically important biologic variants such as postmenopausal changes should be accounted for by the selected reference values.

Only 14 of 52 laboratories reported a specific lower reference value for ferritin in the postmenopausal age group [2]; the reference values were highly inconsistent within the respective ferritin methods and were not consistently adjusted to account for different standardized levels among them, although Roche ferritin methods exhibited relatively higher levels than the compared methods [2,10] (Supplementary Material C). The reference values selected should ensure equal diagnostic efficiency regardless of the provider.

Glycohemoglobin, hemoglobin A1c, is well standardized [11] and its upper reference value was given as 6.4%. The values did not allow for a margin between the reference value and the diagnostic threshold for diabetes, which is defined as a hemoglobin A1c of 6.5% (48 mmol/mol) and above. Such a distinction is recommended because glucose and hemoglobin A1c levels rise well before the clinical onset of diabetes, and the distinct stages of type 1 diabetes can be identified [12].

A precise diagnosis of renal insufficiency depends on accurate biochemical measurements. Despite international endorsement of the standardized creatinine method (isotope dilution mass spectrometry), the upper reference value varied markedly. Clinicians would likely calculate the estimated glomerular filtration rate (eGFR) value manually because GFR values are easier to interpret [13]. In one case, the differing upper reference values for a woman were close to 2-fold (Table 2). It has been considered that the analytical bias and total error of creatinine in serum should be less than 5% and 10%, respectively, although more stringent values have been proposed [14]. Given the CKD-EPI formula [15], a +10% bias of creatinine level in serum would lower eGFR by approximately 10%—falsely increasing the proportion of patients with renal insufficiency by 87.7% (95% confidence interval 81.5 to 94.2%) given the primary health cohort from our database (year 2021, *n* = 86,665; 20–79 years, median 56 years; 55% women). The prevalence of stage 3a chronic kidney disease and above was 5.6%. When reviewing pregnant women, it is crucial to use the standardized creatinine method to sensitively recognize renal insufficiency using trimester-adjusted creatinine limits. Evaluation by eGFR is not recommended in pregnancy [16].

Regarding albumin in serum, the lower reference value differed substantially, but the upper reference value did not. It could be reasoned that different methods with proportional deviation were used, or local decisions were made. Typical biases between albumin methods ranging from 0.3 to 0.6 g/dl [3 to 6 g/l] have regularly been reported, which may lead to unfortunate clinical decisions [17,18,19,20]. Given a bias of +0.5 g/dl (5 g/l) in albumin levels for evaluating multiple myeloma risk groups, 22.5% (confidence interval 16.7% to 29.6%) of the patients could have their prognostic International Staging System (ISS) outcome changed to a better stage solely attributed to the biased albumin measurement (assessment conducted at the Department of Haematology, Oslo University Hospital, Norway, *n* = 160).

## 6. Discussion

Inherently biased clinical assessment introduced by inconsistent organization of biochemical tests, non-methodological routines, inferior standardization, harmonization, and calibration of test methods, and skewed reference values is particularly destructive because repeated testing does not ameliorate the undesirable effect. Biased interpretation of test results stems from systematic deviation from the true (or accepted) value, or from deviation of reference values from the underlying biological distribution.

Scholarly grouping of biochemical tests using a system organ classification system could enhance biological understanding and clinical assessment. A system organ approach is well established in clinical examination, and system organ classes are also used for registration, documentation, and monitoring of pharmaceuticals, vaccines, and drug–device combination products by the *Medical Dictionary for Regulatory Activities* (Supplementary Material A) [21]. In this respect, a systematic organ-based classification of biochemical tests would augment their use by defining indispensable and cost-effective tests for each item. The grouping has biological and clinical meaning and could be used as a rational and mnemonic list that contributes to safety. Departments may choose to reorder items specific to their specialty, although this may entail a risk of unfamiliarity for the general user. Furthermore, the grouping and sorting may be rearranged in departments where the daily focus is on certain items. For example, in intensive care units, blood-gas and acid–base could be grouped with oxygen transportation items (co-oximetry), renal function, and associated electrolytes, and IT solutions could facilitate systematic and competent presentation in different monitoring situations. The organization of the laboratory report should not depend on hospital, chemical, or laboratory preferences or traditions. Organization of biochemical tests according to a system organ classification could add to improved judgment, reading, and overall safety. The motivation for such an organization is ordinary clinical practice.

One important objection to a biochemical system organ classification approach is that most tests relate to different organs and functions. However, this is not different from clinical judgment. For example, chest pain in sickle cell disease may be due to acute chest syndrome, a leading cause of morbidity and mortality. Thus, the examination should not be limited to regular heart items [22]. Additionally, using systematic biochemical reasoning, the system organ classification would increase the biological understanding of chest pain by assessing possible pathology affecting the bone marrow, coagulation cascade, immune system, lungs, heart, and circulation. Well-organized treatment plans based on system organ assessment rely on adjustments for the individual patient that are optimized for diagnostic efficiency, type of practice, and available resources and may therefore differ substantially.

The interval given by the lower and upper reference values is supposed to reflect where 95% (in some cases, 90%) of a healthy population is located—the reference population. Harmonized analytical quality and consequently common reference values of basic tests, such as hemoglobin in whole blood, glycohemoglobin (hemoglobin A1c) in whole blood, and creatinine in serum, could be achieved because they have trustworthy international standards, which are also obtainable for ferritin and albumin [4,11,13,23]. Important stratifications to consider are sex, age from infancy to old age, time, biological cycles, pregnancy, and especially some genetic and phenotypic variants. The most important determination is to study ethnicity as a possible modifying factor. These results cannot be obtained in small local reference studies. The basis for common reference values is internationally standardized or harmonized methods, the creation of which is a long, ongoing task that should be intensified [23,24,25]. Currently, clinical laboratories increasingly use tests provided by a limited number of companies providing equivalent tests internationally. Despite equivalent methods, laboratories may state clearly different reference values [2]. It is difficult for clinicians to discern whether differences between the reference values provided are owing to methodological differences, local calibration, the group of reference individuals chosen, a diagnostic cut-off value, local procedural determinations, or simply the laboratory’s preference. Furthermore, when interpreting a patient’s result, it is challenging for the clinician to recognize potential modifications of the reference value, such as those related to age or values optimized for therapy and follow-up. These variables should be defined and regarded by the user independent of the laboratory used. The reference values provided for interpretation should be familiar and recognizable so that clinicians can efficiently search for pathology.

The biological reference values are vulnerable to both imprecision and bias. The uncertainty of the distribution’s tails may be considerable due to small sample sizes, the sampling procedure of persons to the reference group, and measurement imprecision, and this is the location where sick people’s values tend to occur (Supplementary Material D). Removing outliers through statistical consideration to force the distribution to a certain statistical distribution does not clarify any biological property, such as the occurrence of some important genotypes and phenotypes. Furthermore, bias between methods may be significant and is hardly known to the clinicians, as well as being impossible for them to correct for (Supplementary Material C).

A contrasting situation arises when a diagnostic cut-off value, such as the ferritin level indicating depleted iron stores, is used as the reference value in a laboratory report. This creates a significant risk of clinicians misinterpreting the threshold as a reference value from a normal population. Waiting until iron stores are fully depleted before flagging the ferritin result can put postmenopausal women at an unnecessarily high risk [26]. A case finding of bleeding cancer highlights the importance of settled reference values being understood by all users. Therefore, reference values should ultimately be common throughout the medical community, enabling physicians to cope with variants in their own practice such as age, contraceptive use, or diet.

Biological reference values should be clearly separated from threshold values (cut-off values) which are determined from studies that estimate sensitivity and specificity for rule-out and rule-in determinations. The studies forming the basis for biological reference values and conditional threshold values (cut-off values) should be readily available through notes, publications, or reference to the laboratory handbook. Clinicians should be made aware when non-harmonized methods are used as well.

The biological reference values represent point estimates derived from studies on the selected reference population and do not imply limits for the absence of disease. In this regard, it may be unfortunate to use a less-than or greater-than sign as it can be interpreted as the threshold for a healthy state. Furthermore, because biochemical tests may relate to different systems and organs, a result may remain within the reference interval despite the expected change due to opposing alteration in another system organ’s involvement. Therefore, iron deficiency assessed by ferritin measurement may go undetected, as ferritin may increase in the presence of inflammation or liver involvement. In individual cases, context-driven decision support that integrates and weights all relevant test results within a system-organ-based framework may improve clinical interpretation. However, clinical reasoning should always be the primary guiding framework, with tests selected and interpreted accordingly.

“The Concept of reference values” defines reference values (pubmed.gov, MeSH) (or reference limits) as estimates established using locally calibrated analytical methods applied to a set of locally selected reference individuals [27,28,29]. This means that individuals with conditions such as the sickle cell trait or deficiencies of iodine or iron might be included in the reference group, as the prevalence may be significant in some areas and the condition may be left undisclosed. Constructing reference values only relying on a sample size of 120 persons is accepted [29,30]. With this number, it is likely that important biological aspects will go undetected, and bias may be introduced. Reference values can be verified using only 20 samples from a presumed normal population, which again increases the uncertainty [30]. The procedure reinforces the thinking of clinical assessment of biochemical results as being either normal or abnormal.

There are a plethora of studies proposing reference values that are published or non-published with uncertain methodological accuracy and may include a variable reference population. It is impossible to know whether these can be safely applied in local practice. Important diversity must be investigated in appropriate studies addressing clinically meaningful variants, and reference values should be corrected for important age- and sex-adjusted biological shifts such as changes related to different life stages in women. Factors related to ethnicity should be studied separately. Therefore, the basis of reference values should be calculated from a defined reference population relevant for clinical work across regions and countries. The prerequisite for establishing common reference values is satisfactorily harmonized tests so that unbiased clinical practice can be supported. Because clinicians may be unaware that methods have been standardized or harmonized differently, robust protocols covering the relevant measuring interval should be implemented to address inherent biases [31]. In some cases, acceptable harmonization may not be achieved. Then the diagnostic properties must be examined for each of individual tests in question. Authoritative international institutions play a vital role, not least in securing the accuracy of international clinical guidelines. Current laboratory accuracy standards may be insufficient to detect biases that can have significant clinical effects, in addition to unexpected economic consequences [32].

Non-harmonized methods and reference values impede our understanding and teaching of normal and pathological human biology. Similarly, deleterious biases may be introduced through large language models used in artificial intelligence. From the point of view of patients’ safety, it should also be noted that when a result is “flagged” at one hospital but considered “normal” at another, it can cause unnecessary anxiety and self-referrals. Laboratories should provide standardized clinical notes suitable for patients, non-specialists, and specialists, ensuring that the rationale for the biochemical test and its diagnostic properties is readily available.

## 7. Conclusions

Implementing a scholarly grouping of clinical biochemical tests using system organ classification would enhance the biological approach used in clinical practice. The reference values provided should be biologically based, harmonized, common, and readily recognizable to the clinician. Examination of local laboratory data may reveal biased laboratory support.

## Figures and Tables

**Table 1:** Organization of clinical biochemical tests. Illustrative examples were taken from *The New England Journal of Medicine*, which presents comprehensive laboratory tables in its Clinical Cases.

Examples Showing Inconsistent Organization of Biochemical Tests
The hematology section appeared at the bottom (case 31-2022), in the middle (case 18-2021), or at the top of the laboratory table. The ordering of tests within a section varied from issue to issue. When evaluating bone marrow function, the white blood cell count was in some cases separated from the hemoglobin and platelet counts (case 37-2022).
Tests such as reticulocytes, haptoglobin, and ferritin were not included in the hematology section (case 14-2021).
Vitamin B_12_ was positioned between sedimentation rate and C-reactive protein (case 5-2021).
Fibrinogen and D-dimer were separated from other main coagulation tests (case 7-2021).
Glycated hemoglobin was separated from glucose (case 31-2022).
Ionized calcium was separated from total calcium (case 2-2021).
Lactic acid was not listed together with other acid–base tests; this also applied to total carbon dioxide (case 7-2021).
Valproic acid was presented between glucose and 25-hydroxyvitamin D in one case (case 10-2021).

**Table 2:** Consistency of reference values. Illustrative examples were taken from *The New England Journal of Medicine*, which presents comprehensive laboratory tables in its Clinical Cases.

Examples Showing Inconsistent Organization and Use of Reference Values for Biochemical Tests
The lower and upper reference value for hemoglobin in whole blood in one adult woman varied from 11.2–15.7 g/dl to 13.5–17.5 g/dl (76 years, case 1-2021) and was also given as 14.0–18.0 g/dl (41 years, case 23-2021). The reference values for hemoglobin in blood in men were given as 13.5–17.5 g/dl (57 years, case 15-2022) and 12.0–16.0 g/dl (68 years, case 33-2021; 60 years, case 37-2021).
The lower reference value for ferritin in serum for a woman 64 years old was given as 20 µg/l from “This Hospital” (case 14-2021). However, the handbook states the lower reference value for women as 10 µg/l, “all ages” (Massachusetts General Hospital Homepage, accessed 8 January 2021). Furthermore, the lower reference value was given as 8 and 10 µg/l (woman, 80 years, case 11-2022, “Other” and “This Hospital”) and 10 µg/l (woman, 41 years, case 12-2022, both “Second” and “This Hospital”).
The upper reference limit for glycated hemoglobin in whole blood was given as 6.4% (48 years, case 25-2021). However, the definition of diabetes is set as a hemoglobin A1c of 6.5% (48 mmol/mol) and above, not giving space for the possible state of prediabetes.
The upper reference value for creatinine in serum for females varied within one case from 0.8 mg/dl (71 µmol/l) to 1.50 mg/dl (133 µmol/l) (37 years, case 16-2021). The upper reference value given was 1.50 mg/dl (133 µmol/l) in a pregnant woman (26 years, case 2-2021), a young woman (17 years, case 21-2022), and an older woman (76 years, case 15-2021). The upper reference value for creatinine in serum for men was given in one case as 1.2 mg/dl (106 µmol/l) and 1.5 mg/dl (133 µmol/l) (68 years, case 5-2021).
The lower reference values of albumin in serum varied from 3.3 g/dl to 4.0 g/dl, but the upper reference value was 5.0 g/dl in two hospitals for the same patient (woman, 48 years, case 26-2022).

## Data Availability

The patient results database is confidential and will not be shared.

## Supplementary Materials

The following supporting information can be downloaded at: https://www.mdpi.com/article/10.3390/diagnostics16152475/s1. References [33,34] were cited in Supplementary Materials.
