# Supplementary material for: Clinical Reasoning as the Guiding Framework in Medical Biochemical Test Reporting
Source: Diagnostics (Basel). 2026 Aug 5;16(15):2475. doi: 10.3390/diagnostics16152475 (PMC13465052; doi:10.3390/diagnostics16152475)
Supplement: Supplementary file 1 [file diagnostics-16-02475-s001.zip › Supplementary Sections S1-S4.pdf]

Supplementary Sections S1–S4.

Selected basic and extended tests grouped according to a system organ classification. Tests written in *italics* are included in "The third WHO model list of essential in vitro diagnostics (EDL 3)",*WHO Technical Report Series*, 2020;1031. Implicit specimens are whole blood or plasma (serum) or as specified. Specific preanalytical requirements may apply.

SECTION S1. SYSTEM ("HIGH")

System Organ Grouping	Basic tests	Some extended options, as appropriate
Hematology<br><br>bone marrow and coagulation	• *hemoglobin,* erythrocyte mean corpuscular volume (MCV), mean corpuscular hemoglobin concentration (MCH), *red-cell count*, *white-cell count,*<br>*platelet count*	Subitems:<br>• *white-cell types*<br>• *morphology microscopy; in blood slide*<br>• *lactate dehydrogenase*<br>• immunglobulin.monoclonal-electrophoresis, free-light immunoglobulin chains<br>• hemoglobinopathy tests (*sickle cell*, thalassemia)<br>Items for erythrocytes:<br>• *ferritin*, vitamin B_{12}, folate<br>• methylmalonic acid<br>• homocysteine<br>• reticulocyte count<br>• haptoglobin<br>Iron absorption:<br>• *transferrin-saturation*<br>• hemochromatosis genotyping<br>Coagulation:<br>• *prothrombin international normalized ratio*<br>• *activated partial-thromboplastin time*<br>• *fibrinogen*<br>• von Willebrand tests<br>Thrombosis:<br>• *D-dimer*<br>• Leiden mutation, prothrombin mutation, protein C, protein S, antithrombin<br>• phospholipid antibody test
Immune system<br><br>inflammation, infection, and allergy	• *C-reactive protein*<br>• *erythrocyte sedimentation rate*<br><br>• Microbiological investigation: **Separate scheme**	• *immunoglobulin G, A, and M*<br>Multiorgan autoantibody:<br>• cyclic citrullinated peptide antibody, rheumatoid factor antibody<br>• nuclear antibody<br>Allergy<br>• immunoglobulin E<br>• allergen specific immunoglobulin E antibody: **Separate scheme**
Glucose	• *glucose*<br>• *hemoglobin-A1c*	• *oral glucose tolerance*<br>• glutamate decarboxylase antibody<br>• C-peptide<br>• *ketones; in urine*
Hormones<br>(top to bottom)	• *thyrotropin*,<br>thyroxine, triiodothyronine<br>• *calcium total- / free-*	• *prolactin*<br>• thyroid peroxidase antibody<br>thyrotropin receptor antibody

	• vitamin D 25-OH-	• parathyroid hormone<br>• *cortisol*

SECTION S2. ORGAN

System Organ Grouping	Basic tests	Extended options
Nervous System		• *white-cell count*, *white-cell types*, red-cell count*;* in cerebrospinal fluid<br>• hemoglobin, bilirubin; in cerebrospinal fluid<br>• *protein, glucose*; in cerebrospinal fluid<br>• albumin, IgG, IgG.oligoclonal bands electrophoresis; in cerebrospinal fluid
Lungs<br><br>blood-gas/<br>acid-base		• alpha-1-antitrypsin<br>• *blood-gas and acid-base items*<br>**Separate scheme**
Heart and blood vessels	• *cholesterol: total-,<br>low-density lipoprotein-,<br>high-density lipoprotein-*<br>• *triglycerides*<br>• lipoprotein (a)	• apolipoprotein A, apolipoprotein B<br>• b-type natriuretic peptide<br>• *troponin T / I*<br>• creatin kinase MB-type
Gastrointestinal tract	• calprotectin; in feces<br>• *magnesium*	• *hemoglobin; in feces*<br>• tissue transglutaminase (IgA) antibody<br>• lactase genotyping
Liver and pancreas	• *bilirubin total-*<br>• *alanine aminotransferase, aspartate aminotransferase, gamma-glutamyltransferase, alkaline phosphatase*<br>• *lipase /<br>amylase* pancreas type-	• *bilirubin conjugated-*<br>• bile acids
Kidneys and urinary tract	• *creatinine*, *estimated GFR from creatinine*<br>• *urea / blood urea nitrogen*<br>• *sodium, potassium;<br>in plasma (serum)*<br>• *albumin; in plasma (serum)*<br>• *albumin/creatinine ratio; in urine*<br>• *protein/creatinine ratio; in urine*<br>• *protein total-; in urine*<br>• *test strips; in urine (hemoglobin, albumin, protein, leukocyte esterase, nitrates)*<br>• *morphology microscopy; in urine*	• cystatin C, estimated GFR from cystatin C<br>• *phosphate*<br>• *chloride*<br>• *anion-gap*<br>• *uric acid*
Sexes and fertility	• *human choriogonadotropin; in urine, plasma (serum)*	• *progesterone, follicle stimulating hormone, luteinizing hormone, estradiol,* testosterone, sex hormone binding protein
Muscles	• creatin kinase	• myoglobin

SECTION S3. TRANSCRIPTION

System Organ Grouping	Basic tests	Extended options
Genes and inheritance		• *glucose-6-phosphate dehydrogenase*
Tumor associated		• *prostate specific antigen*<br>• carcinoembryonic antigen<br>• *alpha-fetoprotein*<br>• *human choriogonadotropin optimized for tumor-association*

SECTION S4. EXTERNAL EXPOSURE

System Organ Grouping	Basic tests	Extended options
Drug monitoring and exposures		
