# Supplementary material for: Clinical Reasoning as the Guiding Framework in Medical Biochemical Test Reporting
Source: Diagnostics (Basel). 2026 Aug 5;16(15):2475. doi: 10.3390/diagnostics16152475 (PMC13465052; doi:10.3390/diagnostics16152475)
Supplement: Supplementary file 1 [file diagnostics-16-02475-s001.zip › Supplementary_Materials.pdf]

Supplementary Material

A. System Organ Classification for biochemical tests

Comparison between the grouping for biochemical tests and the listing of *System Organ Classes* used by the *Medical Dictionary for Regulatory Activities* (MedDRA) [33]. Terms in brackets were not used; terms in italics were introduced.

1. (Blood and lymphatic system)
   Hematology, bone marrow and coagulation
2. Immune system and *allergy*
3. (Infection and infestations)
4. *Glucose*
5. *Hormones* (endocrine)
6. Nervous system
7. (Eye)
8. (Ear and labyrinth)
9. (Psychiatric)
10. Respiratory (thoracic and mediastinal)
11. Cardiac
12. Vascular
13. Gastrointestinal
14. (Metabolism and nutrition)
15. Hepatobiliary and *pancreas*
16. Renal and urinary
17. *Sexes*, reproductive (and breast)
18. (Pregnancy, puerperium, and perinatal)
19. Musculo- (skeletal and connective tissue)
20. (Skin and subcutaneous tissue)
21. Congenital, familial, and genetic
22. Neoplasms
23. *Exposures and medication*
24. (General disorders and administration site)
25. (Investigations)
26. (Injury), poisoning, (procedures)
27. (Surgical and medical procedures)
28. (Social circumstances)
29. (Product issues)

B. Comparison of sex differences in flagged low ferritin values relative to colorectal cancer incidence rates

TABLE S1. Comparison of the sensitivity between women and men in detecting incident occult blood loss, a key indicator of colorectal cancer, brought to attention by flagging of low ferritin values (from a primary health cohort, manuscript figure 2). The incidence rates of colorectal cancer were from the Norwegian Cancer Register, https:\\www.kreftregisteret.no, year 2022, accessed 5 February 2024. The overall incidence rate in men was higher than in women. Statistical calculations were performed using R (R Foundation for Statistical Computing 4.5.2 and RStudio 2025.09.2, Boston, MA, USA).

**Ratio (0.95 confidence interval) overall flagged low ferritin values (< 10, < 20 or < 30 µg/l) in women compared to men (< 30 µg/l) from an unselected primary health care cohort divided by the colorectal cancer incidence rate**					**Ratio of flagged low ferritin values per colorectal cancer incidence rate in men compared with women**		
**Age, years**	**Women, reference value 10 µg/l**	**Women, reference value 20 µg/l**	**Women, reference value 30 µg/l**	**Men, reference value 30 µg/l**	**Ratio flagged M/W -10 µg/l**	**Ratio flagged M/W -20 µg/l**	**Ratio flagged M/W -30 µg/l**
[40 to 45)	172 (159 to 187)	636 (610 to 660)	1088 (1057 to 1119)	65 (54 to 78)	0.4	0.1	0.1
[45 to 50)	117 (108 to 127)	439 (421 to 457)	743 (722 to 765)	47 (41 to 54)	0.4	0.1	0.1
[50 to 55)	27 (24 to 31)	114 (108 to 121)	211 (202 to 220)	36 (32 to 41)	1.3	0.3	0.2
[55 to 60)	6 (5 to 7)	29 (26 to 32)	68 (64 to 73)	19 (17 to 22)	3.2	0.7	0.3
[60 to 65)	3 (2 to 4)	18 (16 to 20)	46 (43 to 49)	15 (13 to 17)	5.0	0.8	0.3
[65 to 70)	2 (1 to 2)	13 (12 to 15)	32 (30 to 34)	14 (13 to 15)	7.0	1.1	0.4
[70 to 75)	2 (1 to 2)	12 (11 to 13)	28 (27 to 30)	13 (12 to 14)	6.5	1.1	0.5
[75 to 80)	1 (1 to 1)	10 (9 to 11)	25 (23 to 26)	10 (9 to 11)	10.0	1.0	0.4
[80 to 85)	0.4 (0 to 1)	8 (7 to 8)	19 (18 to 21)	9 (8 to 10)	22.5	1.1	0.4
[85+	1 (1 to 2)	9 (8 to 10)	23 (21 to 25)	10 (9 to 12)	10.0	1.1	0.4

C. Inaccuracy of biochemical methods affects medical decisions

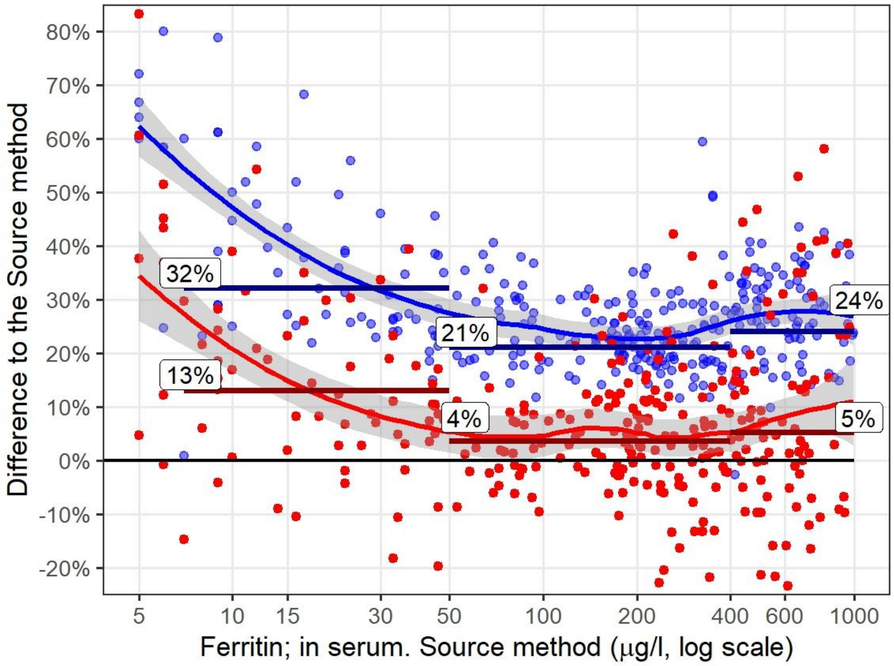

FIGURE S1. Standardization and harmonization in biochemistry are mandatory to achieve consistent clinical results.

The study illustrates the differences between three prevalent internationally used ferritin methods. Because of these differences, relying on a common ferritin value in the lower range to detect bleeding cancer, the hallmark of colorectal cancer, would introduce a substantial biased effect given the large population involved. Similarly, establishing a single threshold for further investigation of hemochromatosis would be meaningless. It cannot be expected that clinicians could decipher different standardized levels or calibrated profiles using a particular method. Because the same method from a company is often used internationally, unaccounted-for bias may influence guidelines. The accuracy of measurements outside the distribution of the reference, or "normal", population is crucial.

The study was conducted using frozen leftover serum, collected and analyzed during 2018 to 2019, n = 312 (Courtesy of the Department of Medical Biochemistry, Sørlandet Hospital Trust, Kristiansand, Norway; Fürst Medical Laboratory, Oslo, Norway; and Stavanger University Hospital, Stavanger, Norway). The methods used were Roche, ECLIA (blue), Abbott, CMIA (red), and Siemens turbidimetry immunoassay (x-axis). The results contained no personal identification and were approved by the hospital's Data Protection Officer. The graphics were produced using R (R Foundation for Statistical Computing 4.5.2 (https://www.R-project.org) and RStudio 2025.09.2, Boston, MA, USA) with the data.table and ggplot2 packages, showing non-linear loess regression (0.95 confidence interval, gray). The bias lines were calculated at clinically relevant concentrations (calculated relative to the average of the compared methods) using Analyse-It for Excel version 5.68.1, Leeds, UK.

D. Accuracy of reference value determination

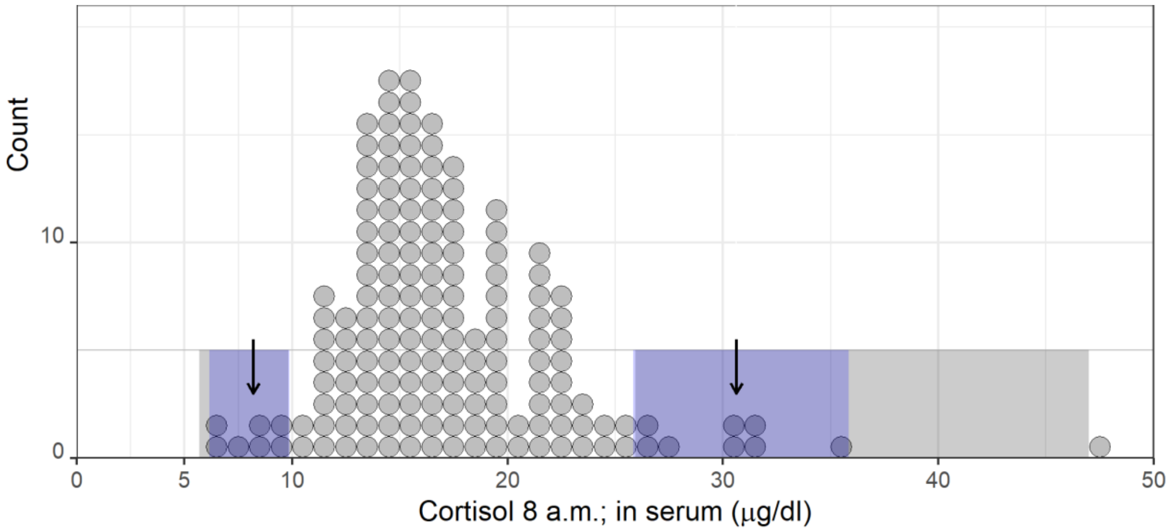

FIGURE S2. A study on the determination of reference values that demonstrate uncertainty at the tails (for illustration purposes).

Cortisol was determined in morning serum samples. Healthy volunteers were recruited (*n* = 160, Caucasian, median age 45 years, interval 21 to 78 years, 54% women). The arrows show the best 2.5- and 97.5 percentile estimates using the non-parametric technique (0.90 confidence intervals are indicated by blue and gray shades, with and without bootstrapping, respectively). Cortisol was measured using the Roche ECLIA method (Department of Medical Biochemistry, Sørlandet Hospital Trust, Kristiansand, Norway). Calculations were performed using "Analyse-It" version 5.68.1 for Excel (Leeds, UK) and "RefVal" [34]. Converting cortisol values: 10 µg/dl = 276 nmol/l.

References

33. MedDRA. Introductory Guide MedDRA Version 29.0 Available online: https://alt.meddra.org/files_acrobat/intguide_29_0_English.pdf (accessed on 25 July 2026).
34. Solberg, H.E. RefVal: A Program Implementing the Recommendations of the International Federation of Clinical Chemistry on the Statistical Treatment of Reference Values. *Comput Methods Programs Biomed* **1995**, *48*, 247–256, doi:10.1016/0169-2607(95)01697-x.
